# Clinical efficacy and safety of acupotomy for acute ischemic stroke: A retrospective study

**DOI:** 10.1097/MD.0000000000049591

**Published:** 2026-07-10

**Authors:** Xiaolei Wu, Yuanyuan Yang, Jiahui Wu

**Affiliations:** a Department of Encephalopathy, Beijing Hospital of Integrated Traditional Chinese and Western Medicine, Beijing, People’s Republic of China.

**Keywords:** acupotomy, acute ischemic stroke, adverse events, Modified Barthel Index, National Institutes of Health Stroke Scale, stroke rehabilitation

## Abstract

Acute ischemic stroke (AIS) is a major cause of persistent neurological impairment and functional dependence. Adjunctive interventions that may enhance rehabilitation-associated recovery warrant pragmatic evaluation in routine clinical settings. This retrospective cohort study consecutively enrolled adults with imaging-confirmed AIS admitted between January 1, 2018 and December 31, 2021. Patients received either standard post-stroke rehabilitation alone (control, n = 61) or standard rehabilitation plus acupotomy (observation, n = 98). Baseline demographics, vascular risk factors, stroke characteristics (including Trial of ORG 10172 in Acute Stroke Treatment [TOAST] classification and infarct territory), and admission status were extracted from electronic records. Primary outcomes were neurological and functional status at discharge assessed by the National Institutes of Health Stroke Scale (NIHSS) and Modified Barthel Index (MBI), with change scores calculated as ΔNIHSS (baseline minus discharge) and ΔMBI (discharge minus baseline). Safety outcomes included procedure-related events, overall adverse events, and serious adverse events. Baseline characteristics were comparable between groups. At discharge, the acupotomy plus rehabilitation group had a lower NIHSS score (4.46 ± 4.05 vs 6.00 ± 4.29; *P* = .026) and greater ΔNIHSS (4.67 ± 2.19 vs 3.22 ± 2.49; *P* < .001). Functional recovery favored adjunctive acupotomy, with higher discharge MBI (76.66 ± 22.85 vs 60.68 ± 25.75; *P* < .001) and greater ΔMBI (28.25 ± 14.05 vs 17.82 ± 10.45; *P* < .001). Procedure-related events were infrequent, and overall and serious adverse event rates were similar. Treatment effects were directionally consistent across prespecified subgroups without significant interaction. Adjunctive acupotomy was associated with improved short-term neurological and functional outcomes without an observed increase in serious safety events in hospitalized AIS patients.

## 1. Introduction

Ischemic stroke remains a leading contributor to mortality and long-term disability worldwide, with sustained growth in absolute case counts despite advances in reperfusion therapies.^[1,2]^ Survivors frequently experience persistent neurological deficits and activity limitations that necessitate structured rehabilitation to mitigate secondary complications and optimize recovery trajectories. Contemporary stroke rehabilitation guidance emphasizes early assessment, individualized goal-setting, and multidisciplinary delivery of task-specific training across the inpatient and post-acute continuum, while recognizing substantial heterogeneity in service availability and intensity. However, real-world rehabilitation “dose” often remains lower than levels suggested by mechanistic and clinical recovery models, and dose delivery is influenced by patient characteristics and health-system factors.^[3]^ The timing of rehabilitation initiation in the acute phase is clinically salient. Very early mobilization and rehabilitation may facilitate functional gains and reduce immobility-related adverse events (AEs), yet the optimal initiation window and the balance between feasibility, safety, and benefit continue to be refined across heterogeneous acute stroke populations.^[4,5]^ In this setting, outcome evaluation typically requires complementary measures that capture both neurological impairment and functional independence. The National Institutes of Health Stroke Scale (NIHSS) is widely used to quantify neurological deficit severity and short-term change, whereas the Modified Barthel Index (MBI) provides a structured appraisal of activities of daily living (ADL) and is commonly used for discharge planning and rehabilitation endpoint assessment.^[6]^

Alongside conventional rehabilitation, integrative interventions are increasingly explored to address post-stroke motor impairment, pain, soft-tissue restrictions, and movement efficiency, particularly in contexts where traditional medicine modalities are routinely incorporated into standard care pathways. Exercise-based rehabilitation strategies and adjunctive approaches remain active areas of investigation, with ongoing efforts to clarify which components yield clinically meaningful incremental benefit beyond usual rehabilitation.^[7]^ Acupotomology (acupotomy), a minimally invasive needling technique incorporating a micro-blade needle, has been used in clinical practice for myofascial and soft-tissue disorders and has recently been evaluated in post-stroke motor dysfunction contexts (e.g., equinovarus foot), suggesting a plausible rehabilitative role that warrants further examination in acute ischemic stroke (AIS) populations.^[8]^

Given the persisting disability burden, variability in rehabilitation delivery, and the need for pragmatic evidence on adjunctive interventions, retrospective comparative studies may provide clinically relevant estimates of effectiveness and safety in routine practice settings.

## 2. Materials and methods

### 2.1. Study design

This study was approved by the Ethics Committee of Beijing Hospital of Integrated Traditional Chinese and Western Medicine. This retrospective cohort study consecutively enrolled patients with a confirmed diagnosis of AIS who were admitted to our institution between January 1, 2018 and December 31, 2021. Eligible patients were allocated according to the rehabilitation strategy documented in the medical records: a control group receiving standard post-stroke rehabilitation alone (n = 61) and an observation group receiving standard rehabilitation plus acupotomy (n = 98). Inclusion criteria were as follows: age ≥ 18 years; AIS diagnosed in accordance with contemporary guideline-based criteria, supported by neuroimaging (computed tomography and/or magnetic resonance imaging) demonstrating cerebral infarction and excluding intracranial hemorrhage; symptom onset to hospital admission within a predefined acute time window (e.g., ≤14 days); stable vital signs permitting initiation of rehabilitation and completion of acupotomy when applicable; and availability of complete baseline clinical data and outcome assessments during hospitalization and/or follow-up.

Exclusion criteria included: hemorrhagic stroke, transient ischemic attack (TIA) without imaging-confirmed infarction, or stroke mimics; severe comorbid conditions likely to confound outcomes or limit survival (e.g., advanced malignancy, end-stage cardiac, hepatic, or renal failure); pre-stroke severe disability (e.g., modified Rankin Scale > 2) or other neurological disorders substantially affecting motor or functional assessment; contraindications to acupotomy or high bleeding risk (e.g., platelet count < 100 × 10^9^/L, uncontrolled coagulopathy, ongoing therapeutic anticoagulation deemed unsafe for needling procedures, or active local infection at the planned intervention site); receipt of other concurrent traditional Chinese medicine interventions specifically targeting neurological recovery beyond routine care, when these could not be reliably documented or adjusted for; and incomplete records or loss to follow-up precluding primary endpoint evaluation. The study was conducted in accordance with the Declaration of Helsinki and was approved by the institutional medical ethics committee. Written informed consent was obtained from all participants or their legally authorized representatives prior to treatment and data use.

The sample size was determined based on the number of eligible patients admitted to our hospital during the study period who met the predefined inclusion and exclusion criteria. Because this was a retrospective observational study, no formal sample size calculation was performed before data collection. Consecutive patients who satisfied the eligibility criteria were included to reduce potential selection bias.

### 2.2. Treatment protocols

Control group protocol: patients received standard post-stroke rehabilitation initiated after confirmation of neurological and hemodynamic stability. The program was delivered by certified rehabilitation therapists and individualized according to baseline deficits. Core components included positioning and early mobilization, passive-to-active range-of-motion exercises, stretching and spasticity management, task-oriented motor relearning, strengthening, transfer training, sitting/standing balance, gait training with assistive devices as needed, and upper-limb functional practice; speech, swallowing, and cognitive rehabilitation were provided when indicated. Rehabilitation sessions were typically conducted once daily, 5–6 days per week, for approximately 40–60 minutes per session throughout the inpatient period (or the documented rehabilitation course). Training intensity was progressively adjusted based on tolerance, fatigue, and cardiopulmonary status, and all sessions were recorded in the medical charts.

Observation group protocol: in addition to the same standard rehabilitation regimen, all acupotomy procedures were performed by licensed physicians with more than 5 years of experience in acupotomy therapy and stroke rehabilitation. To ensure treatment consistency, a standardized treatment protocol was adopted for all patients. Treatment sites were selected according to muscle spasticity distribution, abnormal muscle tension, and functional assessment findings based on rehabilitation evaluation. Before each session, patients underwent safety screening for vital signs, neurological stability, skin integrity at planned sites, and bleeding risk, with treatment deferred in the presence of infection, clinically significant coagulopathy, or unstable status. Acupotomy targeted soft-tissue tension and periarticular restrictions relevant to post-stroke motor dysfunction and spasticity, with conservative depth and direction to avoid neurovascular structures. Each session lasted approximately 10 to 20 minutes, followed by local compression and short observation. Treatments were commonly delivered 2 to 3 times per week for 2 to 4 weeks (or for the documented inpatient course), and rehabilitation therapy was scheduled on the same day when feasible. Procedure-related AEs, including local pain, ecchymosis/hematoma, bleeding, vasovagal reactions, infection, or neurological deterioration, were monitored and documented, and the intervention was discontinued if safety concerns emerged.

### 2.3. Data collection

Clinical data were retrospectively extracted from the electronic medical record system and the institutional stroke registry for all eligible patients admitted between January 1, 2018 and December 31, 2021. A standardized data collection form was used to capture patient-level information from admission through discharge. Collected variables included: demographic characteristics (age, sex, body weight, and body mass index); lifestyle factors (smoking status and alcohol use); vascular risk factors and comorbidities (hypertension, diabetes mellitus, coronary artery disease, atrial fibrillation, prior stroke or TIA, and dyslipidemia); stroke-related clinical characteristics (time from symptom onset to hospital admission and time from admission to initiation of rehabilitation); etiological classification according to the Trial of ORG 10172 in Acute Stroke Treatment (TOAST) criteria and infarct territory (anterior circulation, posterior circulation, or multiple territories) based on neuroimaging reports; and baseline neurological deficit and functional status, assessed using the NIHSS and the MBI at admission or prior to initiation of rehabilitation.

Treatment exposure data were obtained from physician orders, procedural records, and rehabilitation documentation, including assignment to conventional rehabilitation alone (control group) or conventional rehabilitation plus acupotomy (acupotomy group). Outcome measures were collected at discharge, including NIHSS and MBI, and change scores were calculated as ΔNIHSS (baseline minus discharge) and ΔMBI (discharge minus baseline). Safety data were collected from nursing charts, procedure notes, and AE reports, with a prespecified focus on procedure-related events (e.g., local ecchymosis/hematoma, procedure-site pain, minor bleeding requiring compression, local infection, and vasovagal reactions) as well as overall AEs and serious AEs during hospitalization. To enhance data reliability, 2 trained reviewers independently performed data abstraction, and discrepancies were resolved by consensus or adjudication by a senior investigator. All extracted data were de-identified prior to analysis, and completeness checks were performed to identify missing or implausible values; where applicable, source documents were rereviewed to confirm or correct entries.

### 2.4. Statistical analysis

Statistical analyses were performed using IBM SPSS Statistics, version 28.0 (IBM Corp., Armonk). Continuous variables are presented as mean ± standard deviation or median (interquartile range), and categorical variables as number (percentage). Between-group comparisons used the independent-samples *t*-test or Mann–Whitney *U* test for continuous variables, and the chi-square test or Fisher’s exact test for categorical variables. Primary outcomes (discharge NIHSS and MBI, and ΔNIHSS and ΔMBI) were compared between groups using appropriate 2-sample tests. Prespecified subgroup analyses evaluated ΔNIHSS across strata (baseline NIHSS, time window, infarct territory, TOAST), and treatment-by-subgroup interactions were assessed using general linear models. All tests were 2-sided, with *P* < .05 considered statistically significant.

## 3. Results

### 3.1. Baseline demographic and clinical characteristics

Baseline demographic and clinical characteristics were comparable between the control group (n = 61) and the acupotomy plus rehabilitation group (n = 98). Mean age did not differ significantly (67.08 ± 9.10 vs 65.49 ± 9.26 years; t = 1.06, *P* = .290), and the proportion of male patients was similar (57.4% vs 52.0%; χ^2^ = 0.43, *P* = .511). Body mass index and body weight were balanced between groups (both *P* > .05). Smoking status distribution and alcohol use were comparable (*P* = .153 and *P* = .694, respectively). The prevalence of vascular risk factors and comorbidities, including hypertension, diabetes mellitus, coronary artery disease, atrial fibrillation, prior stroke/TIA, and dyslipidemia, showed no statistically significant between-group differences (all *P* > .05). Stroke-related time metrics were also similar, including onset-to-admission time and admission-to-rehabilitation initiation time (both *P* > .05). Etiological classification by TOAST and imaging features (infarct territory, infarct size categories, and large vessel occlusion) did not differ significantly between groups (all *P* > .05). Baseline neurological deficit and functional dependence were comparable, as reflected by NIHSS and MBI scores (*P* = .358 and *P* = .243, respectively; Table 1).

**Table 1:** Baseline demographic and clinical characteristics of the study population.

Variable	Control (n = 61)	Acupotomology (n = 98)	Test statistic	*P* value
Age, yr	67.08 ± 9.10	65.49 ± 9.26	*t* = 1.06	.290
Male sex, n (%)	35 (57.4)	51 (52.0)	χ^2^ = 0.43	.511
Body mass index, kg/m^2^	24.00 ± 2.80	24.04 ± 3.04	*t* = −0.09	.925
Weight, kg	63.57 ± 9.04	63.97 ± 9.57	*t* = −0.26	.793
Smoking status, n (%)			χ^2^ = 3.75	.153
Never	37 (60.7)	54 (55.1)		
Former	5 (8.2)	19 (19.4)		
Current	19 (31.1)	25 (25.5)		
Alcohol use, n (%)	16 (26.2)	23 (23.5)	χ^2^ = 0.15	.694
Hypertension, n (%)	46 (75.4)	68 (69.4)	χ^2^ = 0.67	.412
Diabetes mellitus, n (%)	14 (23.0)	23 (23.5)	χ^2^ = 0.01	.940
Coronary artery disease, n (%)	14 (23.0)	27 (27.6)	χ^2^ = 0.42	.519
Atrial fibrillation, n (%)	16 (26.2)	15 (15.3)	χ^2^ = 2.86	.091
Prior stroke/TIA, n (%)	13 (21.3)	12 (12.2)	χ^2^ = 2.33	.127
Dyslipidemia, n (%)	28 (45.9)	45 (45.9)	χ^2^ = 0.00	.998
Onset-to-admission time, h	8.19 (5.83–11.93)	8.94 (6.33–12.09)	*U* = 2897	.746
Admission-to-rehabilitation initiation, d	1.96 (1.55–2.67)	2.08 (1.55–2.82)	*U* = 2822	.555
TOAST classification, n (%)			χ^2^ = 4.76	.190
LAA	22 (36.1)	29 (29.6)		
CE	18 (29.5)	21 (21.4)		
SVO	16 (26.2)	29 (29.6)		
Other/undetermined	5 (8.2)	19 (19.4)		
Infarct territory, n (%)			χ^2^ = 1.37	.503
Anterior circulation	44 (72.1)	64 (65.3)		
Posterior circulation	15 (24.6)	27 (27.6)		
Multiple territories	2 (3.3)	7 (7.1)		
Infarct size, n (%)			χ^2^ = 0.28	.868
Small	28 (45.9)	41 (41.8)		
Moderate	26 (42.6)	44 (44.9)		
Large	7 (11.5)	13 (13.3)		
Large vessel occlusion, n (%)	9 (14.8)	23 (23.5)	χ^2^ = 1.78	.183
Baseline NIHSS score	9.24 ± 4.34	8.61 ± 3.98	*t* = 0.92	.358
Baseline MBI score	44.08 ± 27.04	49.12 ± 25.17	*t* = −1.17	.243

CE = cardioembolism, LAA = large-artery atherosclerosis, MBI = Modified Barthel Index, NIHSS = National Institutes of Health Stroke Scale, SVO = small-vessel occlusion, TIA = transient ischemic attack, TOAST = Trial of ORG 10172 in Acute Stroke Treatment classification.

### 3.2. Primary outcome comparison

At discharge, neurological and functional outcomes differed significantly between groups. The acupotomy plus rehabilitation group had a lower discharge NIHSS score than the control group (4.46 ± 4.05 vs 6.00 ± 4.29; *t* = 2.26, *P* = .026). Consistently, the magnitude of neurological improvement, quantified as ΔNIHSS (baseline minus discharge), was greater in the acupotomy group (4.67 ± 2.19 vs 3.22 ± 2.49; *t* = −3.72, *P* < .001). Functional recovery assessed by the MBI also favored adjunctive acupotomy: discharge MBI scores were higher in the acupotomy group (76.66 ± 22.85 vs 60.68 ± 25.75; *t* = −3.97, *P* < .001), and the improvement in ADL function (ΔMBI) was larger (28.25 ± 14.05 vs 17.82 ± 10.45; *t* = −5.35, *P* < .001; Table 2).

**Table 2:** Primary outcomes at discharge.

Outcome	Control (n = 61)	Acupotomology (n = 98)	Test statistic	*P* value
NIHSS at discharge	6.00 ± 4.29	4.46 ± 4.05	*t* = 2.26	.026
ΔNIHSS (baseline − discharge)	3.22 ± 2.49	4.67 ± 2.19	*t* = −3.72	<.001
MBI at discharge	60.68 ± 25.75	76.66 ± 22.85	*t* = −3.97	<.001
ΔMBI (discharge − baseline)	17.82 ± 10.45	28.25 ± 14.05	*t* = −5.35	<.001

MBI = Modified Barthel Index, NIHSS = National Institutes of Health Stroke Scale.

### 3.3. Safety outcomes

Procedure-related minor events were infrequent overall. In the acupotomy plus rehabilitation group, local ecchymosis occurred in 6 patients (6.1%), compared with 0 patients (0.0%) in the control group (Fisher’s exact test, *P* = .083). Local hematoma (4.1% vs 1.6%, *P* = .650), procedure-site pain (9.2% vs 3.3%, *P* = .206), minor bleeding requiring compression (4.1% vs 0.0%, *P* = .299), and vasovagal reaction/syncope (6.1% vs 1.6%, *P* = .252) were numerically more frequent in the acupotomy group, but none reached statistical significance. No local infection was observed in the acupotomy group (0.0% vs 3.3% in controls; *P* = .146). The overall incidence of any AE did not differ significantly between groups (28.6% vs 21.3%; χ^2^ = 1.04, *P* = .309). Serious adverse event (SAE) rates were low and comparable (3.1% vs 4.9%; *P* = .676), indicating no detectable between-group difference in severe safety outcomes (Table 3).

**Table 3:** Safety outcomes.

Safety outcome	Control (n = 61)	Acupotomology (n = 98)	Test statistic	*P* value
Local ecchymosis, n (%)	0 (0.0)	6 (6.1)	Fisher	.083
Local hematoma, n (%)	1 (1.6)	4 (4.1)	Fisher	.650
Procedure-site pain, n (%)	2 (3.3)	9 (9.2)	Fisher	.206
Minor bleeding requiring compression, n (%)	0 (0.0)	4 (4.1)	Fisher	.299
Local infection, n (%)	2 (3.3)	0 (0.0)	Fisher	.146
Vasovagal reaction/syncope, n (%)	1 (1.6)	6 (6.1)	Fisher	.252
Any adverse event, n (%)	13 (21.3)	28 (28.6)	χ^2^ = 1.04	.309
Serious adverse event, n (%)	3 (4.9)	3 (3.1)	Fisher	.676

AE = adverse event, SAE = serious adverse event.

### 3.4. Subgroup analyses of neurological improvement

Prespecified subgroup analyses were performed to assess the consistency of the treatment effect of acupotomy across clinically relevant strata, using ΔNIHSS (baseline minus discharge) as the primary measure of neurological improvement. When stratified by baseline stroke severity, adjunctive acupotomy was associated with significantly greater ΔNIHSS in patients with mild (NIHSS ≤ 5; *P* = .012) and moderate (NIHSS 6–15; *P* < .001) neurological deficits, whereas no significant between-group difference was observed among patients with severe stroke (NIHSS > 15; *P* = .837), likely reflecting the limited sample size in this subgroup. Stratification by onset-to-treatment time window demonstrated a consistent benefit of acupotomy in both early (≤7 days) and delayed (>7 days) treatment groups, with statistically significant between-group differences observed in both strata (*P* < .05 for each). Similarly, analyses according to infarct territory showed that the magnitude and direction of treatment effects favored acupotomy in both anterior and posterior circulation strokes. In TOAST-based etiological subgroups, significant improvements were observed in the cardioembolic, small-vessel occlusion, and other/undetermined categories, while the large-artery atherosclerosis subgroup showed a nonsignificant trend toward benefit. Importantly, interaction testing revealed no statistically significant treatment-by-subgroup interactions for baseline NIHSS strata, time window, infarct territory, or TOAST classification (all *P* for interaction > .05), indicating that the observed treatment effect was generally consistent across the examined subgroups (Table 4).

**Table 4:** Subgroup analyses for neurological improvement (ΔNIHSS) and *P* for Interaction.

Subgroup factor	Stratum	Control ΔNIHSS (mean ± SD)	Acupotomology + Rehab ΔNIHSS (mean ± SD)	Test statistic	*P* value	*P* for interaction
Baseline NIHSS stratum	≤5	2.59 ± 1.11	4.43 ± 1.93	*t* = −2.83	.012	.449
Baseline NIHSS stratum	6–15	4.52 ± 1.83	6.32 ± 2.27	*t* = −4.97	<.001	
Baseline NIHSS stratum	>15	7.51 ± 1.88	7.68 ± 1.72	*t* = 0.24	.837	
Onset-to-treatment window	≤7 d	4.52 ± 1.98	6.22 ± 2.22	*t* = −3.88	<.001	.669
Onset-to-treatment window	>7 d	4.27 ± 2.28	5.63 ± 2.57	*t* = −2.17	.035	
Infarct territory	Anterior circulation	4.41 ± 2.01	6.14 ± 2.11	*t* = −3.99	<.001	.891
Infarct territory	Posterior circulation	4.26 ± 2.42	5.78 ± 3.01	*t* = −2.13	.041	
TOAST classification	LAA	4.62 ± 2.03	5.51 ± 2.44	*t* = −1.86	.069	.481
TOAST classification	CE	4.13 ± 2.28	6.80 ± 2.00	*t* = −2.36	.027	
TOAST classification	SVO	4.12 ± 1.92	6.17 ± 2.34	*t* = −3.31	.002	
TOAST classification	Other/undetermined	3.58 ± 1.66	6.80 ± 1.90	*t* = −2.69	.030	

CE = cardioembolism, LAA = large-artery atherosclerosis, NIHSS = National Institutes of Health Stroke Scale, SVO = small-vessel occlusion, TOAST = Trial of ORG 10172 in Acute Stroke Treatment, ΔNIHSS = change in National Institutes of Health Stroke Scale score (baseline − discharge).

## 4. Discussion

In this retrospective cohort of patients with AIS treated between January 1, 2018 and December 31, 2021, adjunctive acupotomy in addition to conventional rehabilitation was associated with more favorable short-term neurological and functional outcomes at discharge. Specifically, the acupotomy plus rehabilitation group demonstrated a lower discharge NIHSS score and a larger neurological improvement quantified by ΔNIHSS. Parallel findings were observed for ADL, with higher discharge MBI scores and larger ΔMBI. Taken together, the outcome pattern suggests that adding a structured, procedure-based intervention to a standard rehabilitation pathway may be associated with incremental gains across complementary domains: neurological deficit (NIHSS) and functional independence (MBI). Because NIHSS and MBI reflect distinct constructs – impairment severity versus functional performance – concordant improvements across both scales support the internal coherence of the observed association and reduce the likelihood that a single measurement artifact explained the results.

From a clinical interpretation standpoint, the between-group differences in discharge NIHSS and discharge MBI are potentially meaningful for inpatient management decisions, including discharge planning, rehabilitation intensity selection, and the need for post-acute services. While the present analysis did not include long-term endpoints (e.g., 90-day mRS), discharge status is nonetheless clinically relevant because early recovery trajectories often influence subsequent rehabilitation engagement and the feasibility of timely transitions to structured post-acute programs. Contemporary stroke rehabilitation guidance emphasizes early, coordinated, and individualized rehabilitation planning, including structured assessment and targeted interventions to address motor and functional deficits. This perspective is reflected in major rehabilitation guidance documents, including the 2024 VA/DoD Clinical Practice Guideline for the Management of Stroke Rehabilitation and its published synopsis, which highlight systematic assessment and tailored rehabilitation strategies across the continuum of care.^[9,10]^

Several mechanistic hypotheses could plausibly explain why adjunctive acupotomy might be associated with improved early outcomes when integrated with rehabilitation. Acupotomy is commonly conceptualized as a minimally invasive needling approach combining mechanical release of soft-tissue restriction with targeted stimulation. In the post-stroke context, early motor dysfunction can be compounded by increased tone, myofascial tightness, pain, and restricted joint range, each of which may limit participation in task-oriented training. A procedure that reduces focal soft-tissue resistance and procedure-site pain may facilitate greater tolerance of mobilization, permit higher repetitions within supervised therapy sessions, and improve the quality of movement practice. Although the present study did not directly measure spasticity, range-of-motion, or therapy dose, the direction of the functional benefit (ΔMBI) is consistent with a pathway in which improved movement capacity enables more effective rehabilitation exposure. Evidence from recent controlled studies in stroke-related motor dysfunction provides partial support for these mechanisms: a 2025 randomized controlled trial evaluating ultrasound-guided acupotomy in post-stroke spastic paralysis reported improvements in MBI alongside motor and neurological deficit measures, suggesting that targeted needling approaches can translate into functional gains in selected stroke populations.^[11]^ In addition, a 2025 Neurological Sciences trial of ultrasound-guided acupotomy combined with acupuncture for post-stroke limb dysfunction reported improvements in functional scales over follow-up, reinforcing the plausibility that acupotomy-based interventions can affect functional recovery when integrated into neurorehabilitation programs.^[12]^

The subgroup analyses provide additional context regarding effect consistency. Adjunctive acupotomy was associated with larger ΔNIHSS in mild and moderate baseline NIHSS strata, while no statistically significant difference was detected in the severe stratum. Importantly, the severe subgroup contained few patients, limiting inference precision and increasing susceptibility to type II error. Stratification by onset-to-treatment time window indicated significant benefits in both ≤7-day and >7-day subgroups, supporting a generally consistent effect direction across the examined windows within the inpatient period. Similarly, favorable effects were observed across anterior and posterior circulation strokes and across etiologic categories, with some heterogeneity in statistical significance by TOAST subtype but no significant treatment-by-subgroup interaction. The absence of significant interaction terms suggests that the association between adjunctive acupotomy and neurological improvement did not differ materially across these prespecified strata, although residual confounding within subgroups remains possible in observational designs. Safety findings further support feasibility. Procedure-related events in the acupotomy group were infrequent and were predominantly minor (e.g., ecchymosis, localized pain, minor bleeding requiring compression, vasovagal reactions). No statistically significant between-group differences were observed in the incidence of any AE or SAEs, and severe outcomes were rare in both groups. This profile aligns with contemporary interventional literature suggesting that acupotomy-related complications are generally uncommon when procedures are standardized and performed in appropriate settings, including recent stroke-related trials that emphasize procedural safety and repeatability under ultrasound guidance.^[13]^ Nevertheless, the numerical imbalance in certain minor events underscores the need for standardized AE ascertainment and explicit periprocedural monitoring protocols, particularly when integrating procedural therapies into routine stroke rehabilitation pathways.

Recent evidence on acupuncture-based or related traditional needling interventions for AIS has generally suggested possible benefits on neurological deficits and functional outcomes, while also highlighting limitations in study quality and heterogeneity. A 2024 systematic review and meta-analysis of randomized controlled trials evaluating acupuncture for AIS concluded that acupuncture may be effective and safe, but emphasized the need for higher-quality evidence to strengthen causal inference and reduce bias.^[14]^ Similarly, a 2024 PLOS ONE systematic review and meta-analysis examining acupuncture combined with rehabilitation in ischemic stroke reported improvements in neurological function outcomes and suggested that integrative approaches may influence recovery-related measures, although included trials varied in design and outcome reporting.^[15]^ Beyond AIS-specific endpoints, methodological discussions have also intensified regarding control conditions and blinding in sham-controlled acupuncture trials. A 2025 Frontiers in Neurology systematic review and meta-analysis focused on sham-controlled designs underscored persistent challenges in standardization, reproducibility, and control selection, all of which complicate the interpretation of pooled effects and may lead to divergent conclusions across reviews.^[16]^

Compared with acupuncture, the evidence base for acupotomy in stroke remains smaller and is more frequently positioned in the context of post-stroke spasticity or limb dysfunction rather than acute neurological recovery. A 2025 randomized controlled trial in the Journal of Electromyography and Kinesiology assessed ultrasound-guided acupotomy for post-stroke spastic paralysis and reported improvements in MBI and motor-related measures relative to a comparator acupotomy approach, supporting the feasibility of acupotomy-based strategies for functional domains relevant to rehabilitation.^[11]^ In a separate 2025 Neurological Sciences randomized study, ultrasound-guided acupotomy combined with acupuncture improved limb function scales and ADL measures over follow-up relative to a basic regimen, suggesting that acupotomy may contribute to functional recovery when layered onto standard care.^[12]^ The present results extend this evolving literature by focusing on an AIS cohort and by evaluating the association of adjunctive acupotomy with discharge NIHSS and MBI alongside improvement metrics (ΔNIHSS, ΔMBI). While direct comparability is limited by differences in populations (acute versus subacute/chronic deficits), intervention content (acupotomy alone versus combined modalities), and outcome timing, the directionality of benefit across neurological and ADL outcomes is consistent with the broader integrative rehabilitation literature in stroke.^[14,15]^

However, because this study was a retrospective single-center observational study without random allocation, selection bias and residual confounding could not be completely avoided. Therefore, the observed associations between acupotomy combined with rehabilitation therapy and improved neurological outcomes should be interpreted cautiously and should not be considered causal. Future multicenter randomized controlled trials are needed to further validate these findings. The sample size was determined by the number of eligible patients treated during the study period rather than by an a priori sample size calculation. Therefore, the statistical power may have been limited, and larger prospective studies are needed to validate the findings.

Although baseline characteristics between groups were generally comparable, this study primarily relied on univariate statistical analyses and did not adjust for important potential confounders such as baseline NIHSS score, onset-to-treatment time, vascular risk factors, and comorbidities. Therefore, residual confounding may have influenced the observed outcomes. Future studies using multivariate regression or propensity-score methods are warranted. In addition, this study only evaluated short-term outcomes at discharge using NIHSS and MBI scores. Standard long-term stroke outcome measures, such as the 90-day modified Rankin Scale (mRS), were unavailable due to incomplete follow-up data. Therefore, the long-term efficacy of acupotomy combined with rehabilitation therapy remains uncertain.

## 5. Conclusion

In this retrospective study, adjunctive acupotomy combined with conventional rehabilitation was associated with superior short-term recovery in AIS. Compared with rehabilitation alone, the acupotomy group showed lower NIHSS scores at discharge and greater neurological improvement (ΔNIHSS), alongside higher discharge MBI scores and larger gains in ADL (ΔMBI). Procedure-related AEs were infrequent, and overall and SAE rates were comparable. Subgroup analyses suggested broadly consistent benefits without significant interaction effects.

## Author contributions

**Conceptualization:** Xiaolei Wu, Yuanyuan Yang, Jiahui Wu.

**Data curation:** Xiaolei Wu, Yuanyuan Yang, Jiahui Wu.

**Formal analysis:** Xiaolei Wu, Yuanyuan Yang, Jiahui Wu.

**Funding acquisition:** Xiaolei Wu.

**Investigation:** Xiaolei Wu.

**Writing – original draft:** Xiaolei Wu.

**Writing – review & editing:** Xiaolei Wu.
